# Fucoidan with three functions extracted from *Sargassum aquifolium* integrated rice-husk synthesis dual-imaging mesoporous silica nanoparticle

**DOI:** 10.1186/s12951-022-01430-9

**Published:** 2022-06-22

**Authors:** Zui-Harng Lee, Meng-Feng Lee, Jung-Huang Chen, Min-Hsuan Tsou, Zhi-Yuan Wu, Cheng-Zhang Lee, Yu-Ya Huang, Showe-Mei Lin, Hsiu-Mei Lin

**Affiliations:** 1 Department of Bioscience and Biotechnology, National Taiwan Ocean University, Keelung, 202 Taiwan; 2 Institute of Marine Biology, National Taiwan Ocean University, Keelung, 202 Taiwan

**Keywords:** *Sargassum aquifolium*, Fucoidan, Rice husk, Mesoporous silica nanoparticles, Anticancer, Target therapy, Dual-imaging

## Abstract

In this study, we used the nanoparticle delivery system to reduce the side effect of conventional cancer treatment- radiation therapy and chemotherapy. We used rice husk silicon source mesoporous silica nanoparticle doped in Eu^3+^ and Gd^3+^ as the carrier in the delivery system and to enable fluorescence and MRI dual-imaging functions for follow-up therapy. In addition, we choose a popular seaweed extract—fucoidan was extracted from the same brown algae—*Sargassum aquifolium* collected from Taiwan-Pingtung-Kenting-Chuanfan Rock. In this research, we used acid hydrolysis to prepared two different molecular weight fucoidan, the small molecular fucoidan (Fus) as drug, and the molecular weight approximately 1 kDa fucoidan (Ful) as the nanoparticle gatekeeper, and as targeting molecule for overexpressed P-selectin on the surface of the metastatic tumors. The results of the cell cytotoxicity experiment showed that HCT116 cancer cells have a survival rate of approximately 58.12% when treated with 200 μg/mL fucoidan. Dual-imaging rice husk mesoporous silica nanoparticles (rMSN-EuGd) were modified with 1 kDa fucoidan (Ful) as the gatekeeper and target, and the small molecule fucoidan (Fus) was loaded into nanoparticles (Ful-Fus@rMSN-EuGd) at a concentration of 200 μg/mL. The HCT116 cancer cells had a survival rate of approximately 55.56%. The cell cytotoxicity experiment results show that Ful-Fus@rMSN-EuGd can improve the anticancer effect of fucoidan, and the nanoparticle drug delivery system using fucoidan as a drug, target, and gatekeeper was successfully synthesized.

## Introduction

Cancer has always been the one of the leading causes of death globally, and cancer treatment methods include chemotherapy [1], surgical resection [2], radiation therapy [3], and nanoparticle drug delivery systems [4]. However the conventional treaments can leading the cancer cell apoptosis but the radioactive and chemotherapy agents are nonselective and can also damage healthy normal tissue. Therefore the bio-accessibility of drugs to tumor tissues is relatively poor, required higher doses of drug also increase the toxicity in normal cell and incidence of drug resistance [5]. To reducing the side effect and increase the therapeutic efficacy, nanoparticle drug delivery systems can use targeting systems to deliver drugs in vivo and to act on specific areas while using passive targeting, called the enhanced permeability and retention effect (EPR effect) [6], or active targeting [7]. Because of the high expression of certain receptors on cancer cells relative to normal cells, modifying the functional molecules on the drug carrier's surface (such as folate [8], hyaluronic acid [9], and fucoidan [10]) can cause them to target cancer cells actively. The commonly overexpressed receptors on cancer cell membranes are the folate receptor, CD44, and P-selectin. In this study, fucoidan acted as the functional ligand target of P-selectin on the cancer cell membrane.

Taiwan is an island surrounded by the sea, which provides a lot of algae resources for algae research [11–13]. During the spring season, there are many brown algae growing in the intertidal zone of Taiwan, with colder areas growing sea tangles and warmer water growing sargassum [14, 15]. Sargassum is a large brown alga that produces fucoidan, sodium alginate, carotenoids, and sargassum polyphenols. Fucoidan has anticancer and anti-inflammatory activities, and sodium alginate has anti-cystic fibrosis and antihypertensive effects [16]. This research focused on using *Sargassum aquifolium* collected from Chuanfan Rock in Kenting-Taiwan to carry out the experiments. There are various extraction methods used to produce fucoidan, such as the acid extraction method [17], enzyme hydrolysis method [18], and hot water extraction method [19–21], and we used the acid extraction method to extract and hydrolysis into small molecular fucoidan (Fus) as the drug in this study.

In metastatic tumor cells, P-selectin is overexpressed [10], and poor P-selectin is overexpressed in normal tissues [22]. Fucoidan can be used to target overexpressed P-selectin on cancer cells [23]. To prevent early drug release during circulation in the body, scientists can modify the ligands on the surface of nanocarriers as gatekeepers to control the release of drugs under specific environments, such as pH values [24], enzymes [25], and oxidant-redox reactions [25]. The controlled release of pH value used in the drug delivery system in this study is achieved by first modifying the nanoparticle with ethylenediamine (EDA) and adding Fe^3+^ to make a metal–ligand complex. Fucoidan itself is negatively charged because it contains sulfate. Sulfate (SO_4_^2−^) interacts with the metal–ligand complex formed by EDA to become the gatekeeper of the nanoparticle, and it will decompose due to the H^+^ ion in the acidic environment of the tumor tissue to release the drug from the carrier [26]. In this experiment, the surface of the nanoparticles was modified by large molecular weight fucoidan (Ful) as a target and gatekeeper.

Next, mesoporous silica nanoparticles were used as the combined therapy nanocarriers. To improve the advantages of traditional mesoporous silica nanoparticles, we used recycled agricultural waste rice husk as the silica source to replace tetraethyl orthosilicate. Rice husk is an agricultural waste that cannot easily be decomposed. At present, it is disposed of using a very environmentally friendly incineration method. It contains organic matter such as cellulose and lignin and minerals such as silicon dioxide and trace elements. The ash content of the calcined rice husk obtained afterward is not only 10–20% higher than that of other biomass fuels, but more importantly, silicon dioxide accounts for 80% of the ash [27]. Rice husk mesoporous silica nanoparticles (rMSN) retain all of the advantages of traditional MSN and have a high specific surface area. There are many advantages of rMSN/ MSN: (1) they are easy to modify and many ligands on their surface can be modified, (2) they have a large pore volume that can improve their ability to load drugs, (3) they are not deposited in the body, (4) they are easily biodegraded, and (5) their main structure is silica oxide, and the silanol group can provide a better environment to protect drugs and modify the chemical surface [28].

**Scheme 1:**
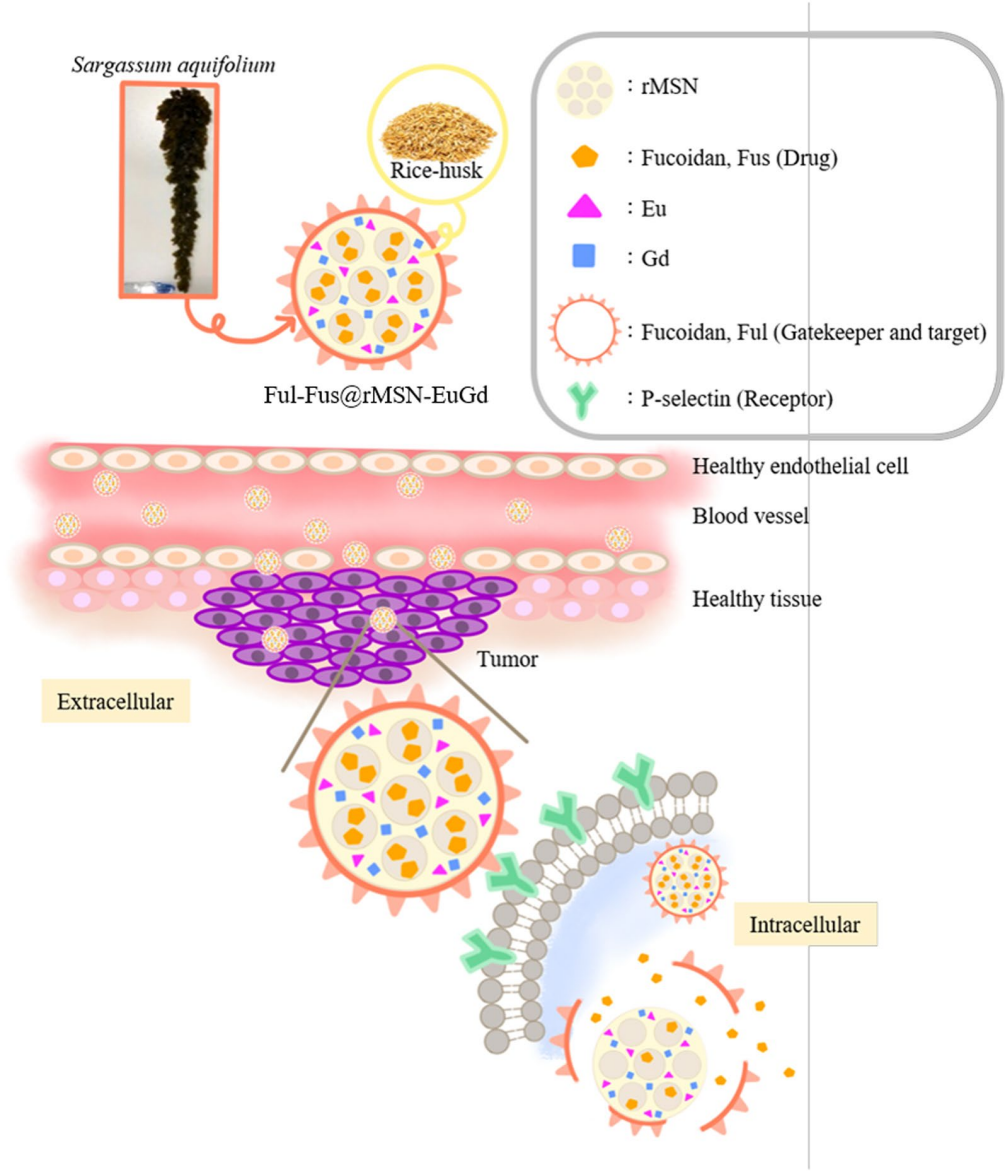
Active transport of Ful-Fus@rMSN-EuGd through the fucoidan targeting and binding to the overexpressed tumor cell receptor (P-selectin) and the H^+^ ion in low pH intracellular environment will dissociation the interaction of fucoidan sulfate between metal–ligand complex cause gatekeeper decompose and the small molecular fucoidan as anticancer drug release toward the cancer cell

This study also used the lanthanide metals europium (Eu) and gadolinium (Gd) to confirm the treatment effect and in vivo location tracking. By doping europium and gadolinium ions into the rMSN structure to archeive red fluorescence [29] and magnetic resonance imaging (MRI) [30] dual imaging effects (Scheme 1).

## Materials and methods

### Materials

98% Tetraethyl orthosilicate (TEOS), (3-Glycidyloxypropyl) trimethoxysilane (GPTMS), ethylenediamine (EDA), 98% Hexadecyl trimethyl ammonium bromide (CTAB), mannose, fucose, glucose, galactose, xylose, McCoy’5A medium and thiazolyl blue tetrazolium bromide (MTT) were purchased from Sigma-Aldrich; 99.9% Europium (III) chloride hexahydrate (EuCl_3_·6H_2_O) and 99.9% Gadolinium (III) chloride hexahydrate (GdCl_3_·6H_2_O) were purchased from Alfa Aesar; Sodium hydroxide (NaOH), Ethanol (C_2_H_5_OH), calcium chloride (CaCl_2_), barium chloride (BaCl_2_), gelatin, hydrochloric acid (HCl), sodium sulfate (Na_2_SO_4_), trifluoroacetic acid (TFA), 1-Phenyl-3-methyl-5-pyrazolone (PMP), methanol (CH_3_OH), chloroform (CHCl_3_), acetonitrile (ACN), sodium nitrate (NaNO_3_), sodium hydrogen phosphate (Na_2_HPO_4_), sodium chloride (NaCl), potassium dihydrogen phosphate (KH_2_PO4) and dimethyl sulfoxide (DMSO) were purchased from J.T. Baker; potassium chloride (KCl) were purchased from Mallinckrodt Chemical; minimum essential medium (MEM) were purchased from Biowes; fetal bovine serum (FBS), non-essential amino acids (NEAA), antibiotic–antimycotic (AA) and trypsin were purchased Gibco; mouse fibroblasts cell (L929) and human colorectal carcinoma cells (HCT116) were purchased ATCC; *Sargassum aquifolium* from Kenting-Chuanfan Rock.

### Algae materials

In this research, the *Sargassum aquifolium* were provided by Lin Showe-Mei Lab of the National Ocean University of Taiwan and collected from Kenting-Chuanfan Rock in March 2018.

After collecting the *S. aquifolium*, it was brought back to the laboratory immediately. After washing it with fresh water, and dried in a large oven at 40 °C, then ground into a powder and sieving by a 35 mesh. After sieved, stored algae powder it in a − 80 °C freezer until later use.

#### Extract of fucoidan

The methods of fucoidan extraction from brown macro algae were as previously research [31]. At room temperature, used 75 mL 95% ethanol mixed 7.5 g algae powder stirred 3 h twice to extract the lipids and pigments, then removed the ethanol and dried the powder overnight.

5 g processed algae powder mixed with 150 mL 0.1 M HCl and stirred 1 h at 42 °C. Used 2 M NaOH to neutralize the mixed solution and collect the supernatant, added double volume 95% ethanol relative to supernatant then freeze-dried the precipitate to obtain crude fucoidan.

Calculated the extraction yeild by using the fomula: (Fa/Fb) × 100%. (Fa: the dry mass weight of the extracted solid; Fb: the dry weight of the algae sample).

#### Chemical analysis of fucoidan

##### Determination of the total sugar content of fucoidan

This experiment uses the phenol–sulfuric acid colorimetric method to estimate the total sugar content of the molecule.

The 10 mg sample (Fucose is the standard) was dissolved in 10 mL of deionized water, 0.4 mL samples were mixed with 20 μL 5% phenol and 1 mL of concentrated sulfuric acid, and then stirred at room temperature 20 min, later measure the 490 nm absorbance.

##### Determination of the sulfate content of fucoidan

This experiment uses the turbidimetric method to estimate the concentration of sulfate in the sample to calculate the content. The principle is the barium ions added to the solution contain sulfate, the barium sulfate will form and the solution becomes cloudy.

First, 0.5 g gelatin was dissolved with 100 mL 60 °C deionized water, added 0.5 g BaCl_2_ into the solution at room temperature, and store at 4 °C. Then 0.1 g sample dissolved in 10 mL HCl and 100 °C refluxed for 1 h, mixed 0.2 mL sample solution with 1 mL BaCl_2_-Gelatin solution for 15 min, and measured the absorbance at 360 nm. For the standard curve, we used sodium sulfate to replace the sample.

#### Fourier transform infrared spectroscopy analysis (FT-IR)

The crude fucoidan was ground with grade potassium bromide (KBr, 1:10) and pressed into a disc under vacuum using a pelletizer. In addition, 0.1 g KBr is used as a background value. The FTIR spectra were obtained using a FTIR spectrophotometer (BRUKER TENSOR Series FT-IR Spectrometer). The infrared spectrum was recorded the wavenumber from 400 and 4000 cm^−1^ and the resolution is 8 cm^−1^, scanning 16 times.

#### Monosaccharide analysis

##### Hydrolysis of fucoidan

The 20 mg sample was mixed with 5 mL 2 M TFA, and hydrolyzed for 4 h at 120 °C. After cooling to room temperature, neutralized the hydrolysate to pH 7 for later subsequent experiments.

##### Derivatization with PMP

At 70 °C water bath, 1 mL of the hydrolysate with 0.5 mL 0.03 M NaOH and 0.05 M PMP-methanol mixed for 60 min. After cooling, the samples with 0.5 mL 0.03 M HCl and then evaporated in vacuo, and then used 1 mL deionized water dissolved. Add 1 mL of deionized water and chloroform to remove excess derivatizing agent. After centrifugation (3500 rpm, 4 °C, 10 min), the supernatant through 0.22 μm membrane filter, the filtrate was collected for HPLC analysis.

##### HPLC determination

The monosaccharides composition of fucoidan determined by high performance liquid chromatography (HPLC) was performed using a Hypersil BDS-C18 column (4.6 mm × 250 mm) at a wavelength of 245 nm. The flow rate was 1.0 mL/min. The monosaccharide mixture containing fucose, xylose, glucose, galactose, and mannose was used as the standard.

#### Molecular weight analysis

##### Hydrolysis of fucoidan

Molecular weight (MW) was determined by using a high-performance liquid chromatogram with a refractive index detector (HPLC-RI) using an OHpak SB-806M HQ column (8.0 mm × 300 mm). Mobile phase: NaNO_3_ (0.1%) solution, the flow rate was 1 mL/min. Then, 1 mg of the sample was dissolved in 1 mL of deionized water and filtered through a 0.22-μm filter. We used pullulan (0.18–1284 kDa) as standards.

### Synthesis of MSN-EuGd

To synthesize MSN-EuGd, 97 mL of deionized water was mixed with 1.4 mL of 1 M NaOH and 1 g of CTAB stirring at 80 °C for 1 h until clear, then add 1 mL of 98% TEOS, 3 mL of 0.05 M EuCl_3_⋅6H_2_O and GdCl_3_⋅6H_2_O were added at 80 °C and stirred. After that, it was washed several times, and finally calcined at 650 °C for 6 h to obtain MSN-EuGd [32].

### Synthesis of rMSN-EuGd

Adding 10% HCl solution to the rice powder and reflux for 48 h, then make the solution neutral, and then dry and calcine to obtain a white powder. 3.8 g of white powder and 1 g of NaOH were added to 38 mL of deionized water, and then refluxed and stirred at 110 °C for one day to obtain a sodium silicate solution (rice husk).

97 mL of deionized water was mixed with 1.4 mL of 1 M NaOH and 1 g of CTAB stirring at 80 °C until clear, then add 3 mL sodium silicate solution (rice husk), 3 mL of 0.05 M EuCl_3_⋅6H_2_O and GdCl_3_⋅6H_2_O were added at 80 °C and stirred. After that, it was washed with water and ethanol several times, and finally calcined at 650 °C for 6 h to obtain rMSN-EuGd [31].

#### Synthesis of GPTMS-rMSN-EuGd

The rMSN-EuGd was added to anhydrous toluene, GPTMS was added dropwise, and then inert gas was continuously introduced at heated 85 °C for 12 h. Then the solution was cooled at room temperature and washed with toluene and ethanol several times in sequence. After drying, GPTMS-rMSN-EuGd was obtained.

#### Synthesis of EDA-rMSN-EuGd

The GPTMS-rMSN-EuGd is added to THF, ultrasonically shaken to disperse, add EDA dropwise, heated to reflux at 45 °C, then cool the solution at room temperature and wash it with ethanol several times, then dry the product to obtain EDA-rMSN-EuGd.

#### Synthesis of Ful-Fus@rMSN-EuGd

The EDA-rMSN-EuGd and Fus were added to deionized water, shaken to disperse, stirred at room temperature, locked several times with deionized water and ethanol, and freeze-dried to obtain the product Fus@rMSN-EuGd. The Fus@rMSN-EuGd to deionized water, then add 24 mM FeCl_3_, stir at room temperature to form Fe^3+^-EDA complex, precipitate the supernatant to obtain the product, wash with deionized water, add Ful, the product was captured with deionized water several times, and then lyophilized to obtain the final product Ful-Fus@rMSN-EuGd.

### Drug release

The EDA-rMSN-EuGd and Rhodamine to deionized water, ultrasonically disperse, and stir at room temperature. After 24 h, wash with deionized water and ethanol for several times, then freeze-dry the product to obtain Rh@rMSN-EuGd. The Rh@rMSN-EuGd add to deionized water mixed with 24 mM FeCl_3_ stir at room temperature to form Fe^3+^-EDA complex. Centrifuge to remove the supernatant to obtain the product, wash with deionized water and ethanol for several times, and freeze-dry the product to obtain the final product Ful-Rh@rMSN-EuGd.

In the experiment, two groups of 20 mg Ful-Rh@rMSN-EuGd were pressed into ingot tablets using an inverting machine, and then put into pH = 7.4 and 5.5 PBS respectively, then shaken evenly, and aspirated 100 μl of the supernatant and transferred to a 96-well plate. Measured the absorbance at 652 nm, and substitutes it into the calibration curve to calculate the total drug release in a cumulative manner.

### Characterization

Use Bruker D2 phase instrument to perform X-ray powder diffraction (XRD) to identify the property of the pore of MSN-EuGd and rMSN-EuGd. Particle size and Zeta potential analysis were performed using dynamic light scattering (Malvern Zetasizer Nano ZS system, Malvern, Worcestershire, UK). Tecnai F30 instrument was used to take transmission electron microscope (TEM) and energy dispersive X-ray (EDX) spectra. Barrett-Joyner-Halenda (BJH) analysis (ASAP 2020, Micromeritics, Norcross, GA, USA) was used for nitrogen adsorption isotherm analysis. The surface area and pore size distribution curves of MSN-EuGd and rMSN-EuGd are determined by Brunauer–Emmett–Teller (BET) method. The BRUKER TENSOR II series spectrometer (Billerica, Massachusetts, USA) was used to confirm the Fourier Transform Infrared (FTIR) spectrum of the characteristic peak of rMSN-EuGd and Ful-Fus@rMSN-EuGd. A Jasco FP-6300 photoluminescence spectrophotometer (Easton, MD, USA) was used to record the luminescence excitation spectrum at an excitation wavelength of 395 nm. Use Netzsch TG 209 F3 equipment to obtain thermogravimetric analysis (TGA) curve to determine the drug loading efficiency when the temperature rises to 700 °C. A 7 T scanner (BRUKER S300 BIOSPEC/MEDSPEC MRI in Karlsruhe, Germany) was used to comfirm the rMSN-EuGd MRI imaging ability. The concentrations of Eu^3+^ and Gd^3+^ ions doped into rMSN were measured by inductively coupled plasma mass spectrometry (ICP-MS, Santa Clara, California, USA) and reported as mass percentages.

### Anticancer activity

#### Cell lines and culture

Normal L929 cells were maintained in MEM medium supplemented with 10% (v/v) fetal bovine serum (FBS) and 1% antibiotics (AA) in an incubator with 5% CO_2_ at 37 ℃. The colon cancer cell line HCT116 was maintained in F-12K medium supplemented with 10% (v/v) fetal bovine serum (FBS) and 1% antibiotics (AA) in an incubator with 5% CO_2_ at 37 °C.

#### Cell viability assay

A normal cell model (L929 cells), and cancer cell models (HCT116 cells) were selected as test cells. The experimental steps were as follows:

For both L929 and HCT116 cells, 2 × 10^4^ per well of cells were seeded in 96-well culture plates, overnight incubated at 37 °C. After overnight incubation, exposed to serial concentrations of materials 4 h, and add 50 μl MTT to each well and incubated for 4 h. Then, add 100 μl DMSO to each well, shake for 15 min, and measured the absorbance values at 540 nm by used enzyme immunoassay analyzer (ELISA reader). The ability of cells to reduce MTT can be used as an indicator of cell viability [31].

The cell survival rate is calculated as follows:

$${\text{Cell viability }} = {\text{ OD}}_{{{54}0}} \left( {\text{test group}} \right)/{\text{OD}}_{{{54}0}} \left( {\text{control group}} \right) \, \times { 1}00 \, \%$$

#### Confocal image analysis

Put the sterilized glass coverslip into a 24-well culture dish. Then planted 2 × 10^4^ cells per well incubate for overnight, each well was added with a mixture of materials and culture medium for co-cultivation, after the completion of the culture, aspirate the culture medium and rinse with PBS twice, then add 3.7% Formaldehyde/PBS solution and react for 10 min. After the reaction is completed, absorb the Formaldehyde solution and rinse with PBS twice, then add 0.1% Triton X100/PBS. After the reaction is completed, absorb the Triton X100 solution and rinse with PBS twice. Stain the cell nucleus with DAPI/PBS and rinse twice with PBS again, place the removed cover slip on the glass slide pre-dropped with mounting glue and mount it on the slide with nail polish, and use Confocal Laser Scanning Microscopy, CLSM (Leica TCS SP5) Observe the phagocytosis of cells and materials.

## Experimental results

### Yield and properties of fucoidan

*Sargassum aquifolium* were collected Pingtung-Chuanfan Rock in March 2018, and then the fucoidan was obtained by acid extraction. As shown in Table 1, it can be seen that the yield was 2.68 ± 0.36%. However, we also found that its molecular weight was 1288.81 ± 62.55 kDa, which indicates a high molecular weight. In 2009, Yoon showed that fucoidan can be divided into low (< 10 kDa), medium (10–10,000 kDa) and high (> 10,000 kDa) molecular weights [33]. This molecular weight falls within the range of (10–10,000 kDa) mid-molecular-weight fucoidan [34], and low-molecular-weight fucoidan has better biological activity and it is easier to develop into anticancer drugs [35]. Therefore, in this study, fucoidan obtained by acid hydrolysis was used. After 1 h of acid hydrolysis, the molecular weight of fucoidan (Ful) was 1.02 ± 0.01 kDa; after 2 h of acid hydrolysis, the molecular weight of fucoidan (Fus) was 0.40 ± 0.01 kDa. We also analyzed the sulfate content by the turbidimetric method, and we observed that the sulfate content of the collected *S. aquifolium* was 35.52 ± 0.25 wt %, since the sulfate content is known to be related to the collection site [36]. To analyze the total sugar content of fucoidan, this study used the phenol–sulfuric acid method. The total sugar content of *S. aquifolium* in Chuanfan rock is 33.20 ± 0.91 wt %.

**Table 1:** Yield, sulfate, total sugar, and molecular weight results of fucoidan

Collection location, number	Yield (%)^a^	Molecular weight (kDa)^a^	MW1 (kDa)^a^	MW2 (kDa)^a^	Sulfate (wt%)^a^	Total sugar (wt%)^a^
Pingtung, Chuanfan Rock	2.68 ± 0.36	1288.81 ± 62.55	1.02 ± 0.01	0.40 ± 0.01	33.52 ± 0.25	33.20 ± 0.91

Molecular weight after acid hydrolysis for 1 h = MW1

Molecular weight after acid hydrolysis for 2 h = MW2

^a^Each value represents the mean ± SD of three determinants of the obtained fractions

To determine the monosaccharide composition of the fucoidans after acid extraction, they were analyzed by HPLC. Table 2 shows that fucoidan contains mannose, glucose, galactose, xylose and fucose. Fucose is the most predominant monosaccharide in fucoidan.

**Table 2:** Monosaccharide composition identification results of fucoidan

Monosaccharide	Mannose (ppm)^a^	Glucose (ppm)^a^	Galactose (ppm)^a^	Xylose (ppm)^a^	Fucose (ppm)^a^
Pingtung, Chuanfan rock	21.28 ± 0.12	51.19 ± 0.34	104.07 ± 0.98	14.89 ± 0.03	176.27 ± 2.24

^a^Each value represents the mean ± SD of three determinants of the obtained fractions.

**Fig. 1:**
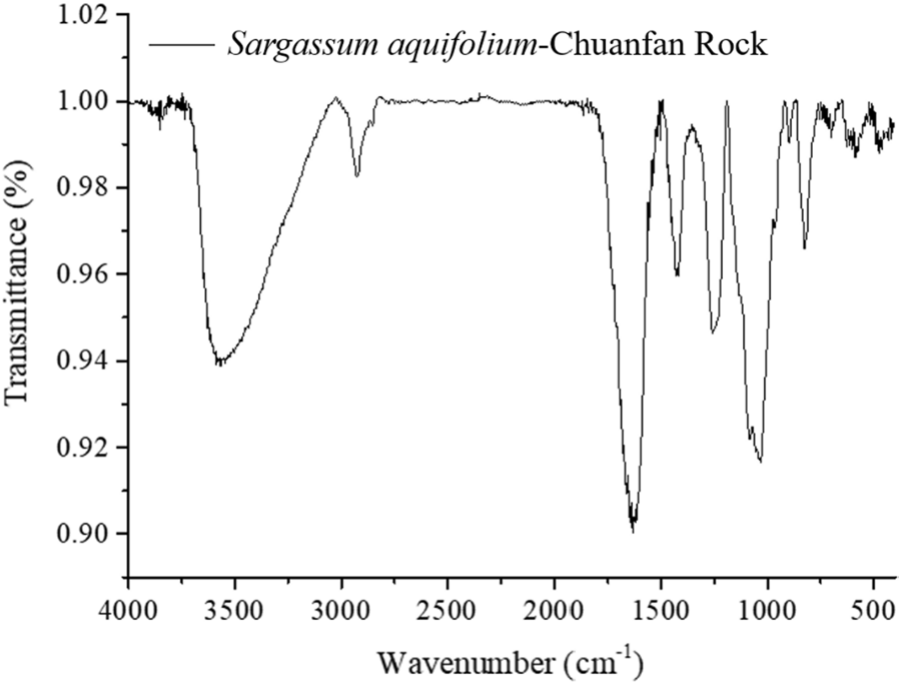
FTIR of fucoidan from Kenting, Pingtung, Chuanfan Rock

The FTIR spectrum of fucoidan extracted by acid extraction is shown in Fig. 1. The peak at 3425 cm^−1^ shows the O–H group of the polysaccharide, the peak at 2960 cm^−1^ indicates the C–H of the carbohydrate, the two peaks at 1641 cm^−1^ and 1420 cm^−1^ are C=O, and the peaks at 1260 and 1056 cm^−1^ are S=O, which are the characteristic peaks of fucoidan and sulfated polysaccharides. The peak at 802 cm^−1^ is C–O–S, confirming the sulfate group, and thus, the results of this study are consistent with the literature [37].

### Structure, formation, morphology, and properties of MSN-EuGd and rMSN-EuGd

**Fig. 2:**
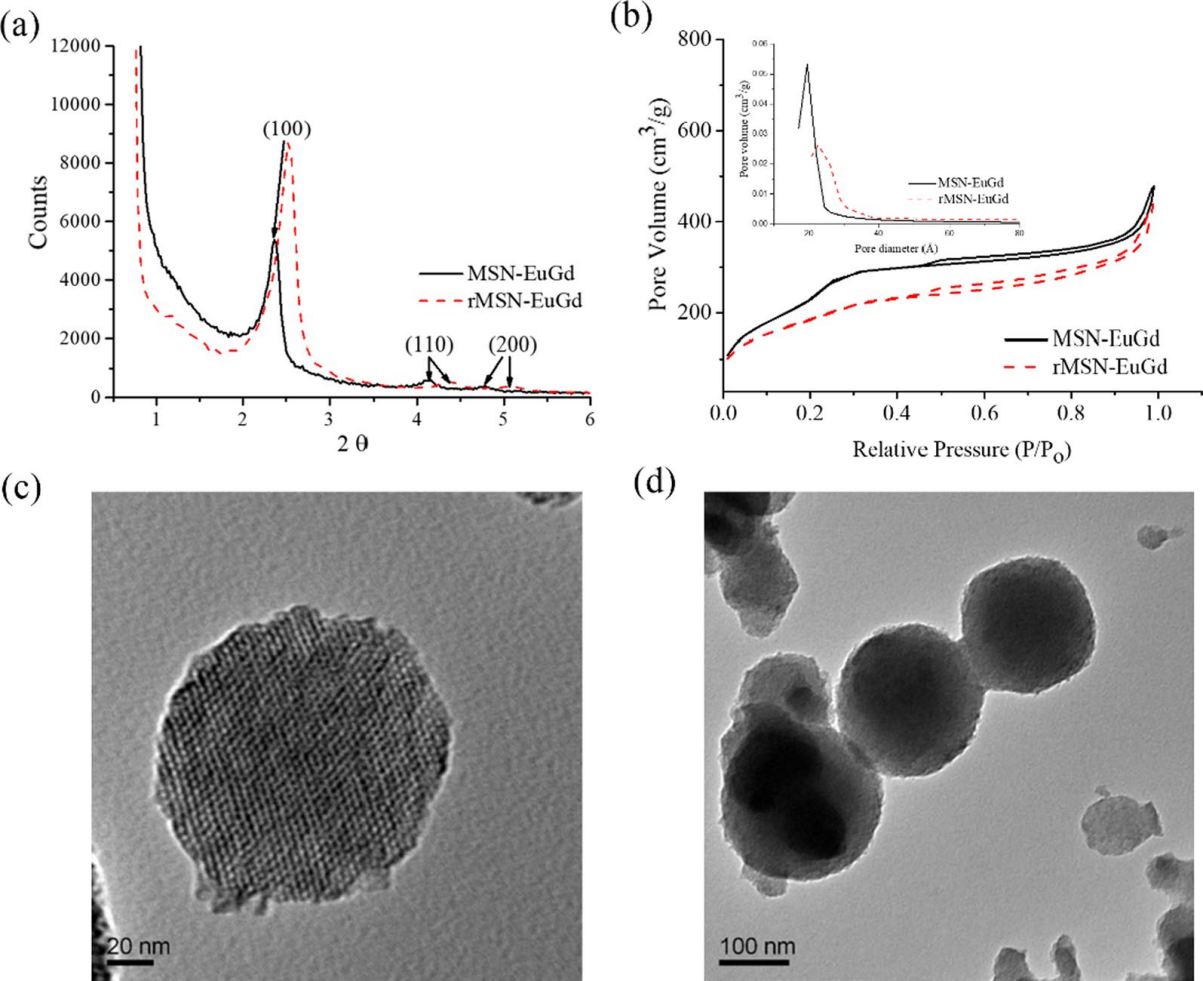
**a** Low-angle XRD patterns of MSN-EuGd and rMSN-EuGd, **b** nitrogen absorption and desorption analysis curves and pore size distributions of MSN-EuGd and rMSN-EuGd, **c** and **d** TEM images of the rMSN structure and size

Figure 2a shows the low-angle XRD patterns of MSN-EuGd and rMSN-EuGd. Both have characteristic peaks at (100), (110), and (200), and the characteristic peak angles of MSN-EuGd are 2.36°, 4.15°, and 4.78°, and those of rMSN-EuGd are 2.52°, 4.78°, and 5.02°. The d-spacing of MSN-EuGd was calculated by XRD and was 3.74 nm, and that of rMSN-EuGd was 3.50 nm. These results show that the materials have a regular hexagonal pore structure [38], and the different silica sources didn’t cause significant differences in the synthesis results.

BET was used to analyze MSN-EuGd and rMSN-EuGd, Fig. 2b shows the nitrogen adsorption and desorption curve, and the pore size distribution pattern. The curve is type IV, indicating mesoporous materials. The pore diameters are 1.95 nm and 2.28 nm, the specific surface areas are 911.04 m^2^/g and 670.18 m^2^/g, and the pore volume are 0.60 cm^3^/g and 0.55 cm^3^/g. The inner wall diameters of MSN-EuGd and rMSN-EuGd are 2.37 nm and 1.76 nm. From Table 3, it can be seen that the pore size and the pore diameter of MSN-EuGd and rMSN-EuGd are not much different, which means that the use of rice husk to synthesize MSNs will not affect the specific surface area, pore size, or pore diameter.

**Table 3:** BET and XRD analysis properties of MSN-EuGd and rMSN-EuGd

Physical data	MSN-EuGd	rMSN-EuGd
XRD 2θ (°)	2.36	2.52
d spacing (nm)	3.74	3.50
BET surface area (m^2^/g)	911.04	670.18
Pore volume (cm^3^/g)	0.60	0.55
BJH desorption diameter (nm)	1.95	2.28
Wall diameter (nm)	2.37	1.76

Through the use of TEM to observe and confirm the structure and size of the nanoparticles [32], Fig. 2c, d show that the rMSNs have a regularly arranged hexagonal hole structure. The pore size is approximately 2–3 nm, which is consistent with the results measured by XRD and BET, while the particle size is approximately 150 nm.

The particle size and surface charge of rMSN-EuGd were measured by DLS. As Table 4, the particle sizes of rMSN-EuGd, GPTMS-rMSN-EuGd, EDA-rMSN-EuGd, Fus@rMSN-EuGd, and Ful-Fus@rMSN-EuGd are 294.2 nm, 356.5 nm, 388.1 nm, 428.7 nm and 431.5 nm, and the surface charge of the materials are − 17.2, − 21.6, + 30.8, − 10.7, and − 28.9 mV. From these data, we know that rMSN-EuGd is negatively charged. When rMSN-EuGd-modified GPTMS, the surface charge changes to − 21.6 [39] and then modifies the EDA, the potential rises to + 30.8 mV because the EDA brings a positive charge [40]. After loading and modifying the fucoidan, since fucoidan is rich in sulfate [26], the potential declines to − 10.7 and − 28.9 mV, which indicates that the fucoidan was successfully loaded and modified to rMSN-EuGd.

**Table 4:** Hydration radius and zeta potential of rMSN-EuGd

	rMSN-EuGd	GPTMS-rMSN-EuGd	EuGd-EDA-rMSN	Fus@rMSN-EuGd	Ful-Fus@rMSN-EuGd
Particle size (nm)	294.2	356.5	388.1	428.7	431.5
Zeta potential (mV)	− 17.2	− 21.6	+ 30.8	− 10.7	− 28.9

**Fig. 3:**
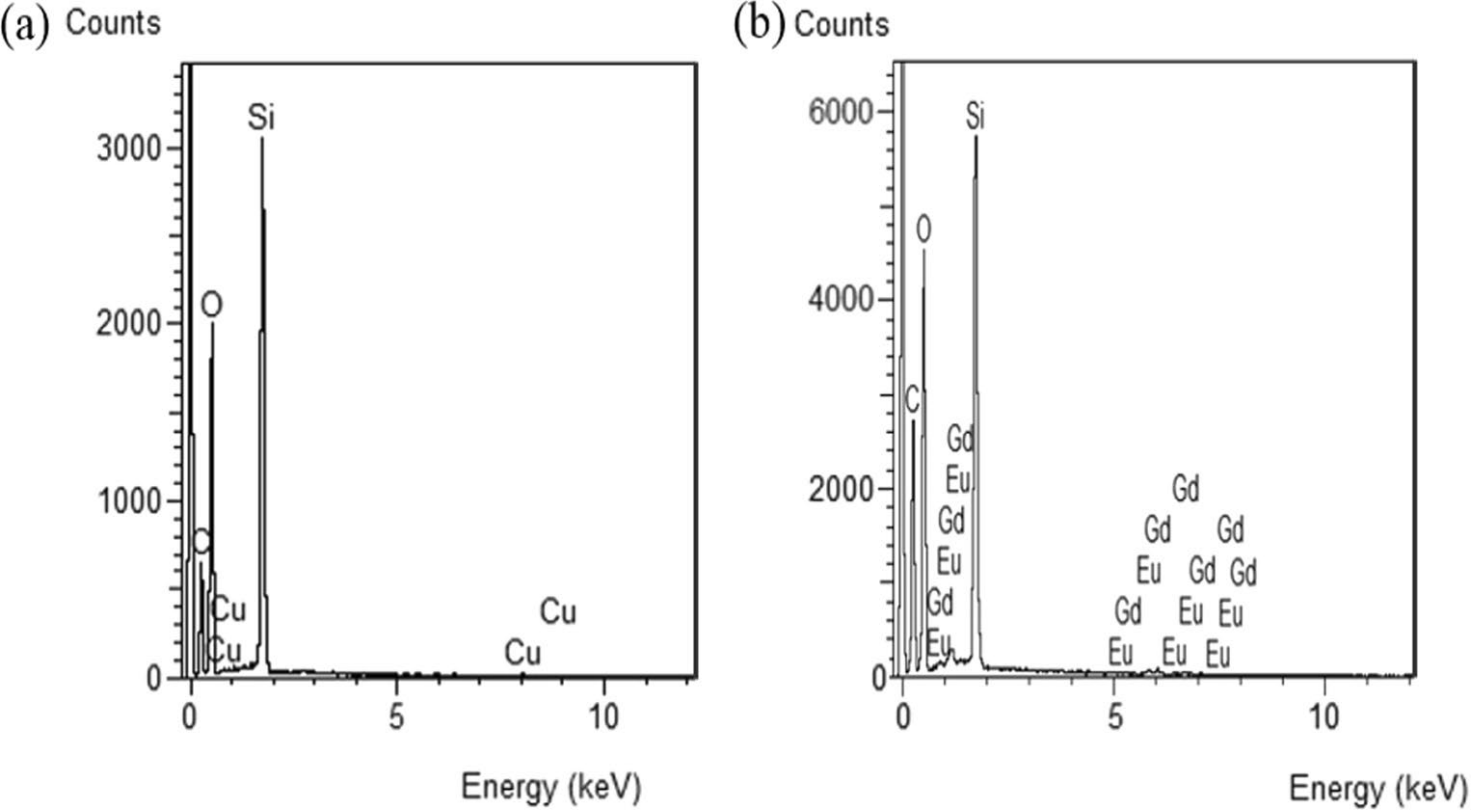
**a** rMSN and **b** rMSN-EuGd elements analysis

Energy dispersive spectroscopy (EDS) was used to analyze the elements contained in rMSNs and rMSN-EuGd. As Fig. 3b shows that rMSN-EuGd has silicon (Si), oxygen (O), europium (Eu), and gadolinium (Gd). By using inductively coupled plasma mass spectrometry (ICP-MS), we can analyze the contents of Eu and Gd in rMSN-EuGd. Table 5 shows that the percentage contents of Eu and Gd were 2.412% and 2.401%, which further proved the existence of lanthanide metal ions.

**Table 5:** ICP-MS analysis of the Eu and Gd percentage contents of rMSNs and rMSN-EuGd

	rMSN	rMSN-EuGd
Eu (%)	0.0	2.412
Gd (%)	0.0	2.401

**Fig. 4:**
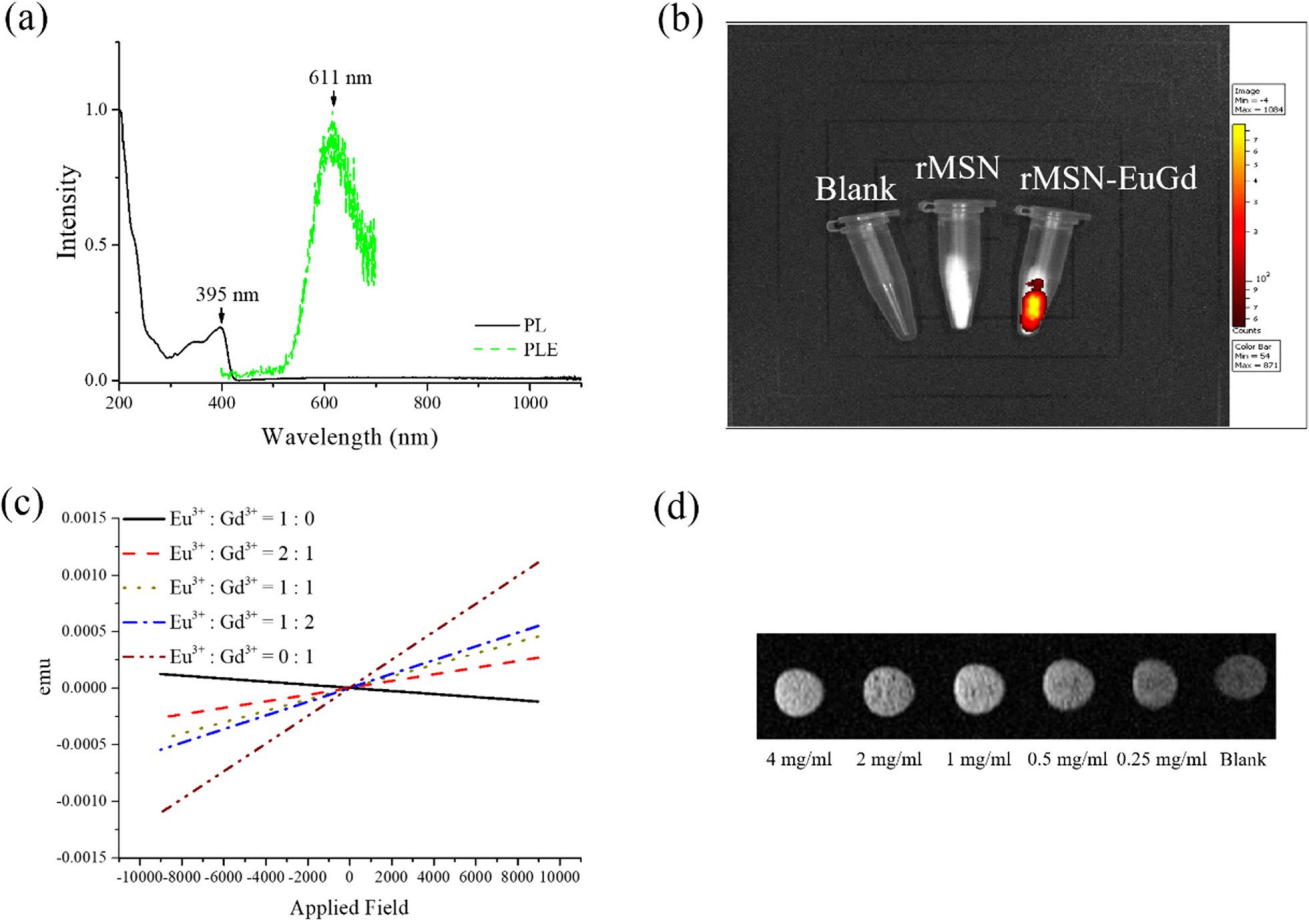
**a** The fluorescence characteristics of rMSN-EuGd; **b** IVIS observes the fluorescence signals of rMSN and rMSN-EuGd at an excitation wavelength of 430 nm; **c** rMSN-EuGd doped with different proportions of Eu^3+^ and Gd^3+^ SQUID analysis chart: **d** T1 MRI image of rMSN-EuGd

A photoluminescence spectrometer (PL), was used to observe the emission characteristics of rMSN-EuGd and the results are shown in Fig. 4a. With 395 nm as the excitation wavelength, an emission peak will be generated at the 611 nm position. Because Eu^3+^ receives 395 nm UV, the energy level will transition from 5D^0^ → 7F^2^ (611 nm) to the red emission wave [41]. If the 611 nm wavelength is used as the emission wavelength, an absorption peak will be observed at 395 nm. These results prove that Eu^3+^ is successfully doped into rMSNs. After confirming the luminescence characteristics of rMSN-EuGd, we used a noninvasive in vivo molecular imaging system (In Vitro Imaging System, IVIS) to observe the luminescence properties of rMSN-EuGd. Figure 4b shows that after we irradiated the sample with a 430 nm excitation wavelength, the blank and rMSNs did not show obvious fluorescence characteristics, while rMSN-EuGd showed very obvious fluorescence characteristics, confirming that rMSN-EuGd can be tracked by IVIS in vivo.

Next, a superconducting quantum interference device (SQUID) was used to confirm whether rMSN-EuGd exhibits Gd^3+^ paramagnetism, and five different Eu^3+^ and Eu^3+^ were synthesized experimentally. Gd^3+^ doping ratio of rMSN-EuGd, Eu^3+^: Gd^3+^  = 1: 0, Eu^3+^: Gd^3+^  = 2: 1, Eu^3+^: Gd^3+^  = 1: 1, Eu^3+^: Gd^3+^  = 1: 2 and Eu^3+^: Gd^3+^  = 0: 1. Five groups, as shown in Fig. 4c, show the changes in magnetism by different Eu^3+^ and Gd^3+^ ratio configurations. As the ratio of Gd^3+^ increases, it tends to become paramagnetic. These experimental results prove that rMSNs doped with Gd^3+^ has paramagnetism.

Because Gd^3+^ is paramagnetic, it is often used as an imaging agent for magnetic resonance imaging (MRI) in medicine. After confirming by SQUID that rMSN-EuGd has paramagnetism, MRI can be used to confirm whether rMSN-EuGd doped with lanthanide metal ion Gd^3+^ can also be used for in vitro MRI. Set the experimental parameters in a 7 T magnetic field, TR/TE = 300 ms, FOV = 7 cm, NEX = 1, slice thickness = 2.00 mm, matrix = 256 × 256, and the material concentration is from 0.25 to 4 mg/mL. Figure 4d shows the MRI T1-weighted image. When the concentration of the rMSN-EuGd material gradually increases, the image shows an increasing brightness trend, which confirms that rMSN-EuGd can use T1-positive imaging for MRI in vitro imaging.

**Fig. 5:**
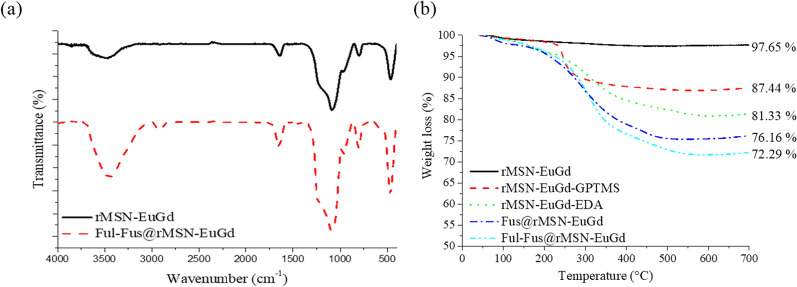
**a** FTIR analysis and **b** TGA curve of rMSN-EuGd, GPTMS-rMSN-EuGd, EDA-rMSN-EuGd, Fus@rMSN-EuGd and Ful-Fus@rMSN-EuGd

Fourier transform infrared spectroscopy (FTIR) was used to analyze the characteristics, chemical compounds, and functional groups of rMSN-EuGd and Ful-Fus@rMSN-EuGd. Figure 5a shows that rMSN-EuGd has a -OH signal at 3443 cm^−1^, Si–O–Si signal at 1100 cm^−1^, and Si–OH signal at 960 cm^−1^ [42]; Ful-Fus@rMSN-EuGd has two peaks at 2934 cm^−1^ and 2877 cm^−1^ due to the stretching vibration signal of the C–H bond, which means that GPTMS [43] is successfully modified. There is a uronic acid C=O absorption peak at 1643 cm^−1^, and there is a strong C–H absorption peak of sugar molecules at 1420 cm^−1^, which proves that the sample contains fucoidan [44].

For determined the surface modification rate and drug loading rate of rMSN-EuGd, GPTMS-rMSN-EuGd, EDA-rMSN-EuGd, Fus@rMSN-EuGd and Ful-Fus@rMSN-EuGd through the thermogravimetric analysis. The analysis results of materials are shown in Fig. 5b. The residual weight percentages are 87.44%, 81.33%, 76.16%, and 72.29%. Through the weight loss percentage in every modified step, the weight loss percentage proves that the organic molecules have been successfully modified, and the fucoidan loading rates is 3.87%, 54.52 mg/g. Compared with other studies [45], the main reason for our low drug loading rate is that in addition to the steric hindrance of the rMSN surface-modified EDA, its positive zeta potential also will attract the negative charge of Fus sulfate, increase the difficult to loaded drug.

### Controlled drug release

**Fig. 6:**
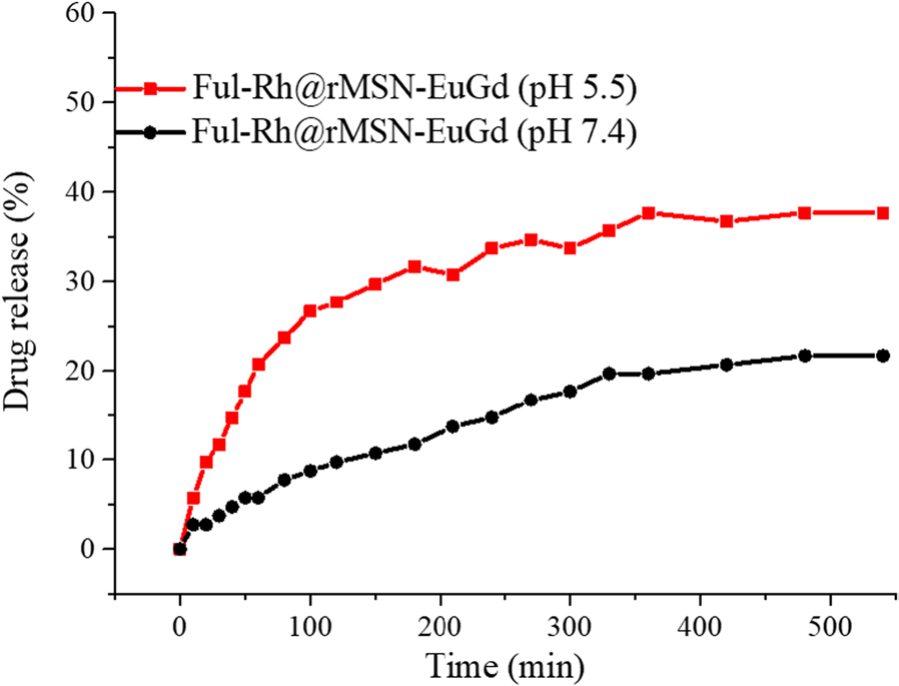
The drug release curve of Ful-Rh@rMSN-EuGd under two PBS environments of pH 7.4 and pH 5.5

To prove that Ful-Fus@rMSN-EuGd can control drug release in different pH value environments, this experiment loaded rhodamine (rhodamine, Rh) instead of Fus and prepared Ful-Rh@rMSN-EuGd. The drug release experiment was conducted in two PBS solutions at pH = 5.5 and pH = 7.4. From the results in Fig. 6, we can see that under neutral environmental conditions of pH = 7.4, the drug release amount of Ful-Rh@rMSN-EuGd is 21.7%, while under the acidic environment of pH = 5.5, the amount of Ful-Rh@rMSN-EuGd drug released is 37.7%, which shows that the design of this experiment can achieve the purpose of controlling the drug release by the pH value.

### In vitro cytotoxicity and cellular uptake of materials

**Fig. 7:**
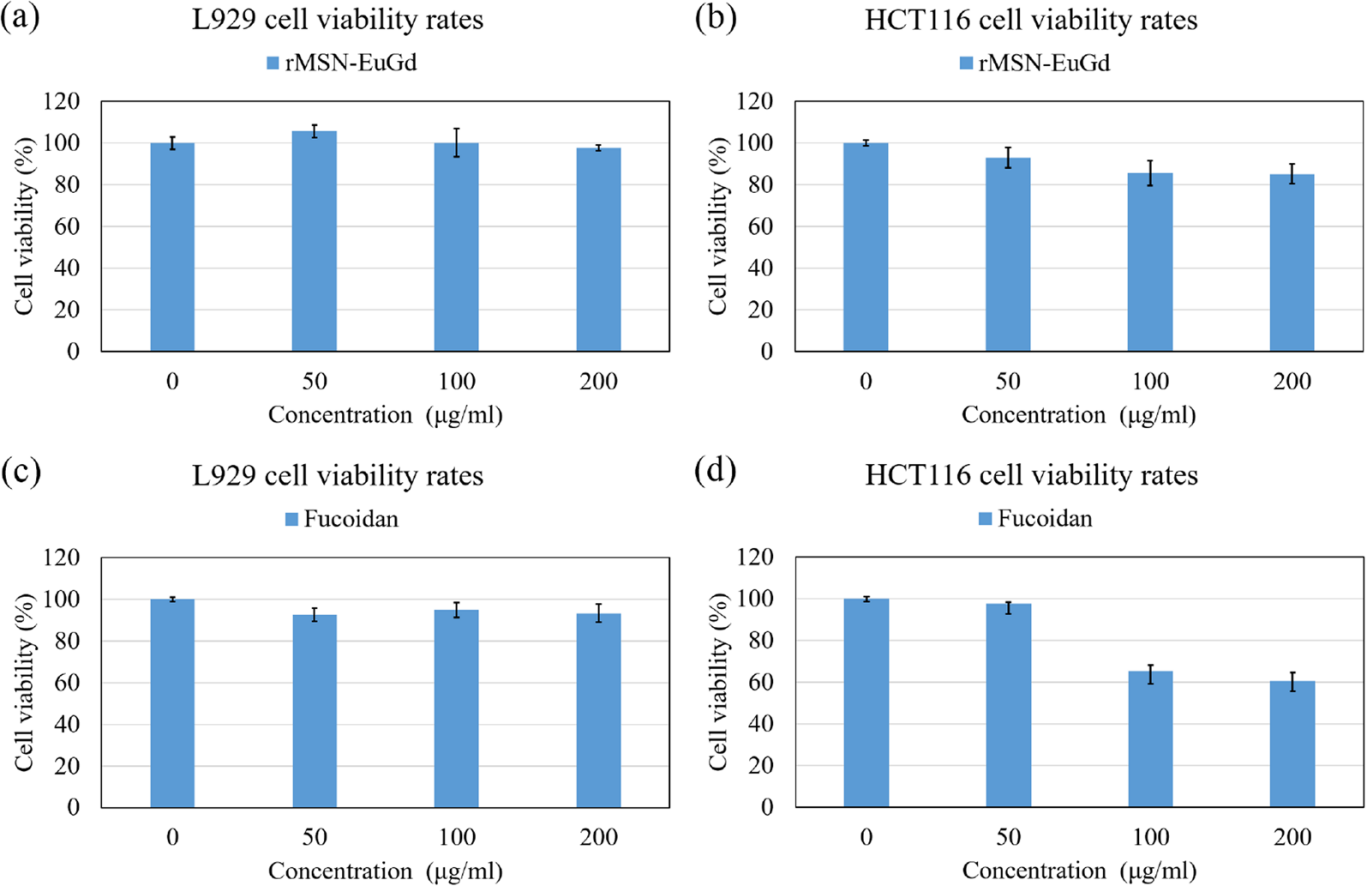
rMSN-EuGd cell viability test for **a** L929 and **b** HCT116 cells; two-hour hydrolysis fucoidan cell viability tests for **c** L929 and **d** HCT116 cells

To determine whether the nanoparticle material has good biocompatibility, this study was conducted by culturing the nanoparticles with mouse fibroblasts (L929) and human colon cancer cells (HCT116) for 72 h to confirm whether the materials were compatible with L929 and HCT116 cells. From Fig. 7a, b, after culturing the L929 and HCT116 cells with rMSN-EuGd for 72 h, the cell survival rate was higher than 80%, which proves that rMSN-EuGd has good biocompatibility and non-cytotoxicity, and the HCT116 cells did not undergo apoptosis due to the influence of the materials.

To verify that fucoidan acid extracted from Taiwan-Pingtung-Kenting-Chuanfan rock collected from *Sargassum aquifolium* is non-cytotoxic to normal cells, Fig. 7c shows fucoidan cultured with L929 cells for 72 h. The L929 survival rate was higher than 80%, show that fucoidan has good biocompatibility with normal cells. In Fig. 7d, the HCT116 cells were cultured with Fus for 72 h, and we calculated that the half-maximal inhibitory concentration (IC_50_) of Fus on HCT116 cells was 6 × 10^2^ µM. The concentration of fucoidan was 200 μg/mL, and the survival rate of the HCT116 cells was 58.12%.

**Fig. 8:**
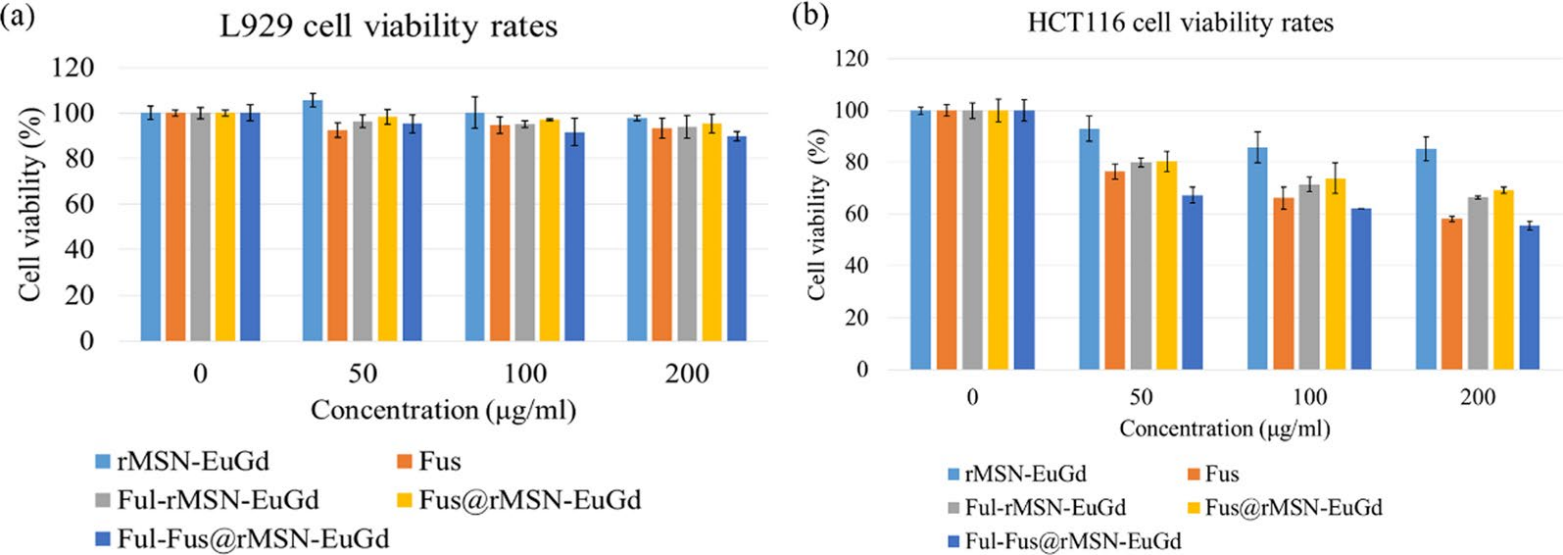
Cell viability of **a** L929 and **b** HCT116 cells

The anticancer activity is affected by many factors, and one of these factors is using a better degradation method to reduce the molecular weight of fucoidan to increase its drug activity [46]. We speculated that the fucoidan extracted from *Sargassum aquifolium* has a relatively low molecular weight after two hours of acid hydrolysis. Most fucoidans with low molecular weights have better anticancer activity than those with high molecular weights [47]. In this study, low molecular fucoidan (Fus) was loaded into rMSN-EuGd as an anticancer drug, and high molecular fucoidan (Ful) was modified on the rMSN-EuGd (Fig. 8).

**Fig. 9:**
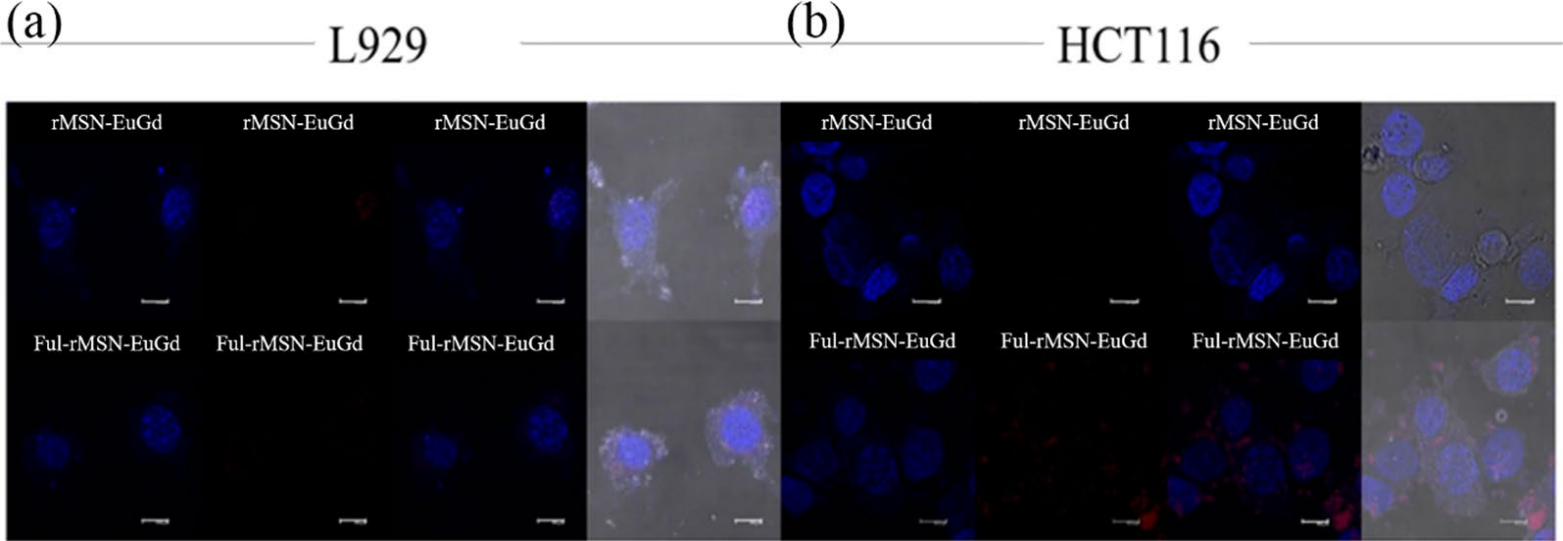
CLSM image of rMSN-EuGd and Ful-rMSN-EuGd cellular uptake of **a** L929 and **b** HCT116 cells

To confirm that the materials can be taken up by cells, this experiment compared the results of L929 and HCT116 cells cultured with rMSN-EuGd and Ful-rMSN-EuGd for 4 h. Figure 9 shows that rMSN-EuGd is rarely taken up by (a) L929 and (b) HCT116 cells, and Fig. 9b shows the signal of Ful-rMSN-EuGd in HCT116 cells, but the signal of Ful-rMSN-EuGd was not significantly detected in L929 cells. Fucoidan modified to rMSN-EuGd targets the high expression of P-selectin on the surface of HCT116 cells. Based on this result, Ful-rMSN-EuGd successfully targeted HCT116 cancer cells and was taken up by the cells.

## Discussion

As the experiment results, we successfully synthesized the rice husk silica source mesoporous silica nanoparticle with dual imaging functions, and compared to other research, the rice husk silica source appears more environmentally friendly. But the size of nanoparticles still needs to improve smaller particles size to increase the cellular uptake [48–50] and get the best composition ratio to synthesize the about 100 nm rice husk silica source mesoporous silica nanoparticle can continue to be discussed.

Previous experiments have focused on combining fucoidan in a therapeutic system to obtain gatekeeper [50], target molecule [51], and anticancer drug [52] functions, but only for one or two fucoidan functions. In this experiment, we are the first person to integrate all fucoidan functions into one therapeutic system but are still affected by the instability of algal extracts.

Therefore, renewable resources and marine active extracts still have a lot of value worthy of research.

## Conclusion

In this study, fucoidan was used as an anticancer drug, nanoparticle carrier gatekeeper, and target molecule. This study used rice husk as biological silica sources and doped Eu^3+^ and Gd^3+^ to synthesize the rice husk mesoporous silica nanoparticles (rMSN-EuGd) for required in vivo fluorescence and magnetic resonance dual imaging functions. Then, fucoidan successfully was loaded into and modified on the surface of rMSN-EuGd as an anticancer drug, gatekeeper, and target molecule. Modified large molecular fucoidan (Ful, molecular weight about 1 kDa) on the surface of the nanoparticles with EDA combined with Fe3 + to form a metal coordination bond complex. From the result of the drug release system, the Ful-Rh@rMSN-EuGd was easily released under the pH 5.5 PBS environment relative to pH 7.4 which proves that the material has a controlled release function.

The cell viability results showed that the survival rate of HCT116 cells was 58.12% when cocultured with 200 mg/mL fucoidan, and 55.56% when cocultured with 200 μg/mL Ful-Fus@rMSN-EuGd. From the TGA results, the amount of fucoidan loaded in 200 μg Ful-Fus@rMSN-EuGd was 10.9 μg/mL, which proves that loading fucoidan into the nanoparticle carrier can achieve better results with a smaller amount of drug. Finally, confocal laser scanning microscopy imaging confirmed that Ful-rMSN-EuGd targets HCT116 cells. The signal of Ful-rMSN-EuGd overlapped with that of HCT116 cells, but there was no such phenomenon in L929 cells, and rMSN-EuGd did not overlap with the HCT116 cell signal, which proves that the surface-modified fucoidan material can target HCT116 cells via targeting the overexpressed P-selectin.

Hopefully, this biosource nanodrug carrier can successfully become a potential delivery material for cancer treatment.

## Data Availability

Not applicable.
